# Validity and Reliability of the Turkish Version of the Temporomandibular Joint Ankylosis Quality of Life Questionnaire (TMJAQoL-TR) in Patients with Severe Temporomandibular Disorders

**DOI:** 10.3390/healthcare14050644

**Published:** 2026-03-04

**Authors:** Manolya İlhanli, Mehmet Alptekin Karaçeşme, Kaan Gündüz, Mahmut Yaran, İlker İlhanli

**Affiliations:** 1 Multidisciplinary Clinics, Faculty of Dentistry, Ondokuz Mayıs University, Samsun 55270, Türkiye; 2 Department of Physical Medicine and Rehabilitation, Physical Therapy and Rehabilitation State Hospital, Samsun 55080, Türkiye; drmehmetalptekin@gmail.com; 3 Department of Dentomaxillofacial Radiology, Faculty of Dentistry, Ondokuz Mayıs University, Samsun 55270, Türkiye; kgunduz@omu.edu.tr; 4 Department of Orthosis and Prosthesis, Faculty of Health Sciences, Ondokuz Mayıs University, Samsun 55270, Türkiye; mahmut.yaran@omu.edu.tr; 5 Department of Physical Medicine and Rehabilitation, Faculty of Medicine, Ondokuz Mayıs University, Samsun 55270, Türkiye; ilker.ilhanli@omu.edu.tr

**Keywords:** quality of life, temporomandibular joint disorders, ankylosis, psychometrics, surveys and questionnaires

## Abstract

Background: The Temporomandibular Joint Ankylosis Quality of Life Questionnaire (TMJAQoL) is a disease-specific instrument designed to assess quality of life in patients with temporomandibular joint (TMJ) ankylosis. No validated Turkish version of this scale existed prior to this study. The aim of this study was to translate, culturally adapt, and evaluate the Turkish version of the TMJAQoL (TMJAQoL-TR) in patients with severe temporomandibular disorders, including a predefined ankylosis subgroup. Materials and Methods: A total of 120 patients with temporomandibular complaints were included. Test–retest reliability was evaluated in a clinically stable subsample of 72 participants with a one-week interval. Following forward–backward translation and cultural adaptation procedures, the TMJAQoL-TR was administered together with the Oral Health Impact Profile Short Form-14 (OHIP-14), the Short Form-36 (SF-36), and Visual Analog Scale (VAS) pain scores. Reliability was assessed using Cronbach’s α, item-level Weighted Cohen’s Kappa, and test–retest Intraclass Correlation Coefficients (ICC), supported by measurement error indices (Standard Error of Measurement [SEM] and Minimal Detectable Change at 95% confidence [MDC_95_]). Construct validity was examined using Spearman correlation coefficients. Structural validity was investigated through exploratory factor analysis, followed by a confirmatory structural model in AMOS to evaluate preliminary model consistency. Floor and ceiling effects were analyzed using the 15% criterion. Results: The TMJAQoL-TR demonstrated excellent internal consistency (Cronbach’s α = 0.879) and very high test–retest reliability (ICC = 0.995; 95% CI: 0.992–0.997). Strong correlations were observed with OHIP-14 (*r* = 0.772, *p* < 0.01), and moderate correlations with VAS pain scores (*r* = 0.312, *p* < 0.01). No significant floor or ceiling effects were detected. A weak but significant negative correlation with the SF-36 physical role subscale suggests that TMJ-related quality of life impairment is associated with role limitations in daily activities, although the magnitude of this association was modest. Exploratory factor analysis supported a clinically coherent two-factor structure, and the AMOS structural model demonstrated acceptable consistency with this framework. Conclusions: The TMJAQoL-TR appears to be a valid and reliable instrument for assessing quality of life in patients with severe TMJ-related functional limitations. Findings from the ankylosis subgroup support potential applicability within the instrument’s original target population; however, further validation in larger ankylosis-specific samples is warranted.

## 1. Background

Following the World Health Organization’s (WHO) definition of health as “a state of complete well-being” within a biopsychosocial perspective, health researchers have increasingly evaluated all areas of health, including oral health, within these dimensions. Oral health is related not only to the physiological characteristics of the mouth, teeth, tongue, and orofacial structures, enabling individuals to perform essential functions such as eating, breathing, and speaking, but also to psychosocial aspects such as self-confidence, well-being, and the ability to socialize and work without pain, discomfort, or embarrassment [1,2]. Over the past 30 years, research has continuously reinforced the strong and growing links between oral health and general health [3]. In light of this relationship, the biopsychosocial model emerged as an alternative to the biomedical approach, which focuses on symptom relief without addressing the “root cause” of the condition [4,5,6]. The biopsychosocial model evaluates individuals’ biological, psychological, and social conditions by analyzing socioeconomic status, educational level, interests, and living conditions together, thus ensuring comprehensive care and multidisciplinary treatment [7].

One of the factors affecting oral health is the range of motion of the temporomandibular joint (TMJ). TMJ range of motion may be impaired due to trauma, infection, or degenerative changes, leading to functional limitation, restriction of mouth opening, and disability [8,9]. TMJ ankylosis can cause limited range of motion, malnutrition, dentofacial deformities and asymmetry, poor oral hygiene, dental caries, speech disorders, chewing difficulties, and obstructive sleep apnea [10,11]. These issues impose both physical and psychosocial burdens on individuals, affecting their quality of life. Although conventional quality of life assessments reveal the impact of these conditions, there is a clear need for a tool specifically designed to assess TMJ ankylosis. While data obtained through the biomedical model remain valid, self-reported perceptions regarding the physical and psychosocial effects of disorders or diseases complement clinical indicators and provide a more comprehensive, multidimensional health assessment for individuals and communities, aligning better with the biopsychosocial model and the International Classification of Functioning, Disability, and Health [9].

Quality of life is defined as “an individual’s perception of their position in life within the context of the culture and value systems in which they live, and in relation to their goals, expectations, standards, and concerns” [2]. Oral health plays a critical role in an individual’s overall health status, quality of life, and social participation. Studies have demonstrated a relationship between oral health and oral-health-related factors with quality of life [12,13]. According to the United Nations’ 2030 Agenda for Sustainable Development, oral health constitutes the first step in achieving the third goal ensuring healthy lives and promoting well-being for all at all ages [14].

Various assessment tools have been developed to evaluate oral health and temporomandibular disorder (TMD)-related quality of life across different dimensions. These include the Oral Health Impact Profile Short Form-14 (OHIP-14) [15], the Orthognathic Quality of Life Questionnaire (OQoLQ) [16], the Oral Behaviors Checklist (OBC-21) [17] and the Temporomandibular Joint Ankylosis Quality of Life Questionnaire (TMJAQoL) [18]. Turkish validity and reliability studies have been conducted for OHIP-14 [19] and OBC-21 [17], but not for TMJAQoL. The distinctive feature of TMJAQoL compared to other questionnaires is that it evaluates quality of life specifically in relation to TMJ ankylosis rather than general oral health [18].

The TMJAQoL was originally developed for TMJ ankylosis; however, it may also be applicable in patients with severe TMD-related functional limitations, as these individuals share similar restrictions in mouth opening, mastication, speech, and social participation. For this reason, the present study included both patients with confirmed TMJ ankylosis and those with severe TMD presenting comparable functional impairment. This sampling strategy allowed for exploratory validation of the Turkish version while retaining the instrument’s condition-specific foundation.

Therefore, the aim of this study was to translate, culturally adapt, and evaluate the validity and reliability of the TMJAQoL-TR in patients with severe temporomandibular disorders, including a predefined subgroup with clinically and radiologically confirmed TMJ ankylosis.

## 2. Materials and Methods

This study was a methodological psychometric validation study designed to evaluate the translation, cross-cultural adaptation, and measurement properties of the TMJAQoL-TR through a cross-sectional assessment with a one-week test–retest reliability component in patients with severe temporomandibular disorders, including a predefined temporomandibular joint ankylosis subgroup.

All participants were informed about the study objectives and procedures, and written informed consent was obtained prior to participation. Patient confidentiality was ensured at all stages. The study protocol was approved by the Ondokuz Mayıs University Clinical Research Ethics Committee (OMUKAEK 2021/115) and conducted in accordance with the Declaration of Helsinki. The study is reported in accordance with the STROBE statement for observational studies.

### 2.1. Materials

This prospective cross-sectional study included 120 consecutive patients who presented to the Ondokuz Mayıs University Physical Medicine and Rehabilitation outpatient clinic between March 2023 and March 2025. The number of patients who participated in the test–retest study was 72 (60%). The mean age of female patients was 51.2 ± 12.1 years (range: 23–81), while the mean age of male patients was 43.2 ± 16.2 years (range: 18–78). The mean age of females was significantly higher than that of males (*p* = 0.004). The demographic characteristics of the patients are shown in Table 1.

Among the total sample, 26 participants (21.7%) were diagnosed with TMJ ankylosis, while 94 participants (78.3%) were classified as having severe temporomandibular disorders without ankylosis. Detailed demographic characteristics are presented in Table 1. Sample size estimation followed the conventional “10 participants per item” heuristic commonly used in validation research. Methodological recommendations indicate that a minimum of 100 participants and approximately 5–10 participants per item are sufficient for preliminary psychometric validation studies [20]. Accordingly, a final sample of 120 participants was considered adequate for this validation phase.

Participants were eligible for inclusion if they were aged 18 years or older, had a clinical diagnosis of temporomandibular disorder or temporomandibular joint (TMJ) ankylosis, provided written informed consent, and were able to understand and independently complete the questionnaire. Participants were excluded if they had a severe, uncontrolled systemic disease, an active psychiatric disorder or cognitive impairment, or were undergoing psychological treatment at the time of enrollment.

A flow diagram summarizing participant recruitment and study phases is provided in Figure S1 (Supplementary Materials).

### 2.2. Methods

TMJ ankylosis was diagnosed based on a combination of clinical examination (marked restriction of mouth opening and lateral excursions) and radiological confirmation (CBCT and/or MRI) showing fibrous or bony fusion of the joint, in accordance with previously published criteria. In non-ankylosis cases, temporomandibular disorders were diagnosed according to clinical criteria broadly consistent with the Diagnostic Criteria for Temporomandibular Disorders (DC/TMD), including pain on palpation, joint sounds, and functional limitation. Participants were classified as having severe TMD-related functional limitation if they demonstrated (a) restricted opening or lateral movements consistent with functional impairment or (b) preserved mandibular range but marked pain-provoked disability affecting mastication, speech, or daily oral functions.

Temporomandibular joint ankylosis was further classified according to the Sawhney classification system, which categorizes ankylosis into four types based on radiographic and anatomical findings. Sawhney’s classification divides TMJ ankylosis into four types based on the extent of movement limitation [21].

- Type 1: The condylar head is visible but severely deformed; fibroadhesions prevent TMJ movement.
- Type 2: The articular surface and the deformed condylar head are mostly fused at the margins and in the anterior/posterior parts, while the medial surface remains intact.
- Type 3: The ankylotic mass involves the mandibular ramus and zygomatic arch; an atrophic and displaced anterior condylar fragment lies medially.
- Type 4: A bony ankylotic mass extends between the mandibular ramus and cranial base, completely obliterating the TMJ.

The study was conducted and reported in accordance with COSMIN guidelines for studies evaluating measurement properties of patient-reported outcome measures (Supplementary File S1). The methodological quality of the study was evaluated according to the COSMIN (COnsensus-based Standards for the selection of health Measurement INstruments) Risk of Bias framework. Each measurement property was rated as “very good,” “adequate,” “doubtful,” or “inadequate,” and overall results were interpreted using COSMIN criteria for sufficiency (+), insufficiency (−), or indeterminate evidence (?).

#### 2.2.1. Assessment Tools

The patient data collection form, OHIP-14, TMJAQoL-TR, and Short Form-36 (SF-36) were used to evaluate the patients. The patient data form recorded name, age, gender, occupation, educational status, comorbidities, duration of TMD complaints, and pain. Additionally, measurements of mouth opening, right and left lateral deviation were taken. OHIP-14, SF-36, and TMJAQoL-TR were used to assess patients’ quality of life.

The OHIP-14 scale evaluates seven dimensions of the impact of oral conditions on people’s quality of life: functional limitation, physical pain, psychological discomfort, physical disability, psychological disability, social disability, and handicap [19]. The statements refer to the past year. The Turkish validity and reliability of OHIP-14 was conducted by Başol et al. [19]. In contrast to general quality of life questionnaires, OHIP-14 is more useful for identifying psychosocial impacts among individuals and groups by focusing more on patient-centered, psychological, and behavioral outcomes [19,22]. In the scale’s evaluation, “never” is scored as 0, “rarely” as 1, “sometimes” as 2, “often” as 3, and “very often” as 4. Higher total scores indicate greater severity of problems and lower quality of life.

The SF-36 is a general tool assessing health-related quality of life over the past four weeks across eight dimensions: physical functioning, physical role, bodily pain, general health, vitality, social functioning, emotional role, and mental health. All items related to each dimension (except for health transition) are summed and transformed to a scale ranging from 0 to 100, where higher scores indicate better health or well-being [23]. The Turkish validity and reliability study was conducted in 2018 [24].

The TMJAQoL is the first quality of life scale specifically designed for patients with TMJ ankylosis. The scale consists of 12 items evaluating four dimensions of quality of life: symptoms, functional limitation, psychological well-being, and social well-being. It possesses optimal psychometric properties for assessing quality of life in patients with TMJ ankylosis. Each item has five response options: “never” (0), “almost never” (1), “sometimes” (2), “quite often” (3), and “very often” (4). Higher scores indicate lower quality of life [18].

Pain intensity was additionally assessed using a 10 cm Visual Analog Scale (VAS), where 0 indicated ‘no pain’ and 10 indicated ‘worst imaginable pain’. The VAS was used to evaluate concurrent validity by examining its correlation with TMJAQoL-TR total scores.

#### 2.2.2. Translation and Cultural Adaptation Process

Written permission was obtained from the original developers of the TMJAQoL to carry out its Turkish validity, reliability, and cultural adaptation. For the translation procedure, the method proposed by Beaton et al. [20] was followed. The translation and cultural adaptation of the TMJAQoL were conducted in six stages:

1. Stage 1—Formation of the Translation Team: A translation team was formed, consisting of one dentist and one physician. Both forward translators were native Turkish speakers fluent in English; one had clinical expertise in temporomandibular disorders, and the other had methodological experience in health measurement instruments. The translators worked independently to minimize bias. The synthesis process focused on semantic, idiomatic, experiential, and conceptual equivalence rather than literal translation. During the expert committee review, discrepancies were resolved through consensus, ensuring conceptual fidelity to the original construct measured by the TMJAQoL.
2. Stage 2—Forward Translation: The original English version of the TMJAQoL was translated into Turkish independently by the two native Turkish-speaking researchers. Each researcher worked separately on the translation.
3. Stage 3—Synthesis of Translations: The two draft translations were reviewed by the same translators. Differences between the drafts were discussed, and a consensus was reached to produce the Turkish version of the TMJAQoL.
4. Stage 4—Back Translation: The Turkish version was back-translated into English by an English linguist with no medical background and by a dentist independently. No conceptual inconsistencies were detected during the back translation; therefore, the translated text was accepted as the Turkish version of the TMJAQoL.
5. Stage 5—Expert Committee Review: The research team compared the Turkish version with the original English version to evaluate for any technical inconsistencies.
6. Stage 6—Pilot Testing: To verify fluency, clarity, and comprehensibility, the Turkish version was administered to 10 patients with TMJ ankylosis and to 5 dentists not involved in the study. Patients in the pilot study were not included in the final sample. Feedback confirmed that the translation was appropriate, and the final version, named TMJAQoL-TR, was approved.

#### 2.2.3. Statistical Analysis

Statistical analyses were performed using IBM SPSS 21.0 (IBM Corp., Armonk, NY, USA). Distributional assumptions were examined using the Kolmogorov–Smirnov test and inspection of skewness and kurtosis values. Variables with *p* > 0.05 and skewness–kurtosis values between −1 and +1 were considered normally distributed. Based on distribution results, parametric or non-parametric statistical tests were selected accordingly. Since several scale scores demonstrated non-normal distribution, correlation analyses were performed using Spearman’s rank correlation coefficient. Descriptive statistics were used to present the demographic and clinical characteristics of the patients.

All questionnaires were reviewed for completeness at the time of administration. No item-level missing data were detected in the final dataset for TMJAQoL-TR, OHIP-14, or SF-36. Therefore, no data imputation procedure was required. In cases where minor omissions occurred during initial completion, participants were asked to review and complete the missing responses immediately. Consequently, analyses were conducted using complete-case data.

#### 2.2.4. Reliability

##### Internal Consistency

Internal consistency measures the degree to which all items in a scale are correlated with each other. High inter-item correlations indicate that the items measure the same construct. This is expressed using Cronbach’s alpha. Values of Cronbach’s alpha range from 0 to 1.0, with 1.0 indicating perfect internal consistency. Values above 0.80 are generally considered acceptable for a scale [25].

##### Test–Retest Reliability

The entire sample did not participate in the test–retest due to reasons such as missed appointments, scheduling issues, and undocumented clinical visits. Test–retest analysis was conducted in a clinically stable subsample of 72 participants; full-sample retesting was not mandatory due to stability/recall considerations. Patients participated in the retest after one week. During this period, no new treatment was initiated, and participants reported no significant change in their symptoms. The second assessment was conducted under standardized outpatient conditions in the same clinical setting, using identical instructions. As the TMJAQoL-TR is a self-administered questionnaire, examiner-related variability was minimized. A 7-day retest interval was chosen to minimize recall bias while avoiding true clinical change in this chronic condition, in line with general psychometric recommendations for test–retest reliability.

For test–retest reliability, the ICC was calculated to analyze the correlation between responses to all items and total scores in both administrations. In scientific studies, ICC values are interpreted as follows:

- <0.20: Poor;
- 0.21–0.40: Fair;
- 0.41–0.60: Moderate;
- 0.61–0.80: Good;
- 0.81–1.00: Very good [26,27,28].

ICC estimates with 95% confidence intervals were obtained using a single-rating, absolute agreement, two-way mixed-effects model. ICC values between 0.60 and 0.80 indicate good reliability, while values above 0.80 indicate excellent reliability [28].

To evaluate item-level stability, Weighted Cohen’s Kappa statistics (quadratic weighting) were calculated for each Likert item in the test–retest dataset. This method accounts for the ordered categorical structure of Likert responses, assigning partial credit for adjacent disagreements. Kappa values were interpreted as follows: <0.40 = poor, 0.41–0.60 = moderate, 0.61–0.80 = good, and >0.80 = excellent agreement [29].

Measurement error was evaluated using the Standard Error of Measurement (SEM) and the Minimal Detectable Change (MDC). Test–retest reliability was calculated with a two–way mixed effects, absolute agreement, single-measure Intraclass Correlation Coefficient (ICC), in accordance with psychometric recommendations for clinical scales. SEM was computed using the formula SEM = SD × √(1 − ICC), reflecting the degree of measurement precision. To determine the smallest amount of change that exceeds measurement error, the MDC at the 95% confidence level (MDC_95_) was calculated as MDC_95_ = 1.96 × SEM × √2. This approach aligns with COSMIN guidelines and current methodological standards, indicating that score changes greater than the MDC_95_ value can be interpreted as true clinical change rather than random variability or measurement error.

Floor and ceiling effects were examined by calculating the percentage of participants who obtained the lowest or highest possible score on the scale. A value ≥15% was considered indicative of a floor or ceiling effect, in line with commonly accepted psychometric criteria.

#### 2.2.5. Construct Validity

Construct validity was assessed using a hypothesis-testing approach. We expected moderate-to-strong positive correlations between TMJAQoL-TR and the OHIP-14 total score, reflecting convergent validity, and weak-to-moderate correlations with VAS pain scores. In addition, we hypothesized negative correlations between TMJAQoL-TR scores and SF-36 subscales related to physical and emotional functioning (physical function, physical role, and emotional role), indicating that poorer quality of life would be associated with lower general health-related quality of life scores.

#### 2.2.6. Exploratory Factor Analysis

Prior to factor extraction, the suitability of the data for factor analysis was assessed. Sampling adequacy was evaluated using the Kaiser–Meyer–Olkin (KMO) statistic, and the strength of the correlations among the items was examined with Bartlett’s test of sphericity. An exploratory factor analysis (EFA) was conducted to investigate the construct validity of the TMJAQoL scale. The factor structure was determined using the Kaiser criterion (eigenvalue >1), and a varimax orthogonal rotation was applied to clarify factor loadings and improve interpretability. Items with factor loadings ≥0.40 were considered acceptable. The factor loading matrix, total explained variance, and eigenvalues were examined for structural consistency, and factor labels were assigned based on conceptual and clinical relevance.

#### 2.2.7. Confirmatory Structural Modeling (AMOS)

A confirmatory structural model based on the exploratory findings was organized in AMOS (IBM SPSS AMOS 23.0, IBM Corp., Armonk, NY, USA) and standardized regression weights, factor covariances, and initial model fit indices (CFI, TLI, RMSEA, χ^2^/df) were reported to preliminarily evaluate structural consistency.

#### 2.2.8. Additional Statistical Analysis

Construct validity of the TMJAQoL-TR was further examined using exploratory factor analysis (EFA) with evaluation of standardized factor loadings (λ), communalities (h^2^), composite reliability (CR), and average variance extracted (AVE). Factor loadings were interpreted as strong (≥0.70), moderate (0.60–0.69), or acceptable (0.50–0.59), and items with loadings below 0.50 were considered for removal. Communality values ≥0.50 were regarded as well explained by the latent construct, whereas values between 0.30 and 0.49 were considered acceptable for clinical measurement instruments. Cross-loading was assessed by examining whether an item loaded on multiple factors with a loading difference <0.20, which would indicate insufficient factorial discrimination. Internal consistency at the construct level was evaluated using composite reliability (CR), with values ≥0.70 considered indicative of good reliability. Convergent validity was assessed using average variance extracted (AVE), where values ≥0.50 indicated adequate variance capture, while values between 0.40 and 0.49 were deemed acceptable in multidimensional clinical scales. These interpretation thresholds follow contemporary recommendations for health measurement instrument validation and structural equation modeling-based psychometric evaluation as outlined by Mokkink et al. [30] within the COSMIN framework, classical latent variable guidance by Fornell and Larcker [31] for AVE and CR interpretation, and general SEM reporting standards described by Kline [32]. These criteria were adopted to ensure that the Turkish adaptation demonstrates not only internal consistency but also adequate representation of latent constructs and factorial distinctiveness in a clinical population.

## 3. Results

All 120 patients included in the study completed the TMJAQoL-TR, OHIP-14, and SF-36 questionnaires. Of the 120 participants, 70.0% (n = 84) had no comorbidity, whereas 30.0% (n = 36) had at least one comorbid condition. Comorbidities are given in Table 2.

Diagnoses were established through clinical examination and/or panoramic radiography and/or Cone Beam Computed Tomography (CBCT) and/or Magnetic Resonance Imaging (MRI). Fourteen patients were diagnosed solely based on clinical examination without imaging. The etiological data of conditions causing TMJ ankylosis are presented in Table 3.

A total of 26 participants were diagnosed with temporomandibular joint ankyloses (Table 3). Regarding medical history, 14 had childhood jaw trauma, 3 had ankylosing spondylitis, 1 had rheumatoid arthritis, and 1 had a psoriatic arthritis. Seven of them had no known etiologic factor.

Imaging findings showed that 17 participants had reduced anterior disk displacement, 9 had non-reduced anterior disk displacement, 1 had an increase in condylar sclerosis, 1 had TMJ erosion with non-synovitis, and a total of 63 participants demonstrated degenerative changes. However, no imaging-related pathology was detected in five individuals who reported TMJ pain. Additionally, clinical evaluation indicated suspected anterior disk displacement in 14 participants who did not undergo imaging. Also, a total of 18 participants were diagnosed with bruxism.

The median duration of symptoms was 60 months (range: 1–360 months). The measurement outcomes and questionnaire scores are presented in Table 4.

### 3.1. Internal Consistency

Cronbach’s alpha of the TMJAQoL-TR was 0.879, which reflects good internal consistency according to established psychometric criteria, suggesting that the scale items are sufficiently correlated and consistently represent the underlying construct.

#### 3.1.1. Test–Retest Reliability

Retest reliability analysis demonstrated robust temporal stability for the TMJAQoL-TR. The overall test–retest Intraclass Correlation Coefficient (ICC) was 0.995 (95% CI: 0.992–0.997), indicating *excellent* reliability. Measurement error indices were also satisfactory, with a SEM of 0.54 and a corresponding MDC_95_ of 1.5 points, suggesting that changes exceeding this threshold can be interpreted as true score variation rather than measurement imprecision. Item-level reliability further supported these findings; all individual items showed “very good” ICC values (Table 4) and no missing responses were observed among the 72 participants included in the retest dataset.

Agreement statistics also confirmed consistent item performance over time. Weighted Cohen’s Kappa values ranged from 0.835 to 0.980, demonstrating *excellent* ordinal agreement and reinforcing the scale’s temporal stability at the item level. Collectively, these psychometric properties indicate that the TMJAQoL-TR provides reliable measurement for both clinical assessment and research applications (Table 5).

For the total score, the SEM was 0.54 and MDC_95_ was 1.5 points, indicating that changes above this threshold can be interpreted as true clinical change rather than measurement error. Consistently, measurement error analysis for the subscales showed SEM values of 0.34 for Factor 1 and 0.41 for Factor 2, with corresponding MDC_95_ values of 0.94 and 1.14 points. Together, these results suggest that changes of approximately ≥1 point in either subscale and ≥1.5 points in the total score can be considered clinically meaningful and beyond random measurement variation (Table 6).

#### 3.1.2. Measurement Error

Floor (4.2%) and ceiling (0.2%) effects remained below the 15% criterion, demonstrating an appropriate distribution of scores without clustering at the extremes.

### 3.2. Construct Validity

The correlation between the TMJAQoL-TR questionnaire and the OHIP-14 was found to be high (*r* = 0.772; *p* < 0.01), while a moderate correlation was observed between the TMJAQoL-TR and the VAS pain scale (*r* = 0.312; *p* < 0.01). Additionally, a moderate and statistically significant correlation was found between the OHIP-14 and the VAS pain scale (*r* = 0.370; *p* < 0.01). A good correlation was identified between the amount of right and left mandibular deviation (*r* = 0.710; *p* < 0.01). The mean maximal incisal opening was 43.7 ± 9.2 mm, with no significant correlation with TMJAQoL-TR scores.

The VAS pain scale was negatively correlated with the SF-36 physical role subscale (*r* = −0.324; *p* < 0.01) and positively correlated with the SF-36 pain subscale (*r* = 0.333; *p* < 0.01).

The SF-36 physical function subscale showed a positive correlation with the SF-36 physical role subscale (*r* = 0.619; *p* < 0.01) and a negative correlation with the SF-36 pain subscale (*r* = −0.584; *p* < 0.01).

The SF-36 emotional role subscale was positively correlated with physical function (*r* = 0.372; *p* < 0.01) and physical role (*r* = 0.561; *p* < 0.01) while being negatively correlated with pain (*r* = −0.361; *p* < 0.01).

Correlation results between the assessment scales are presented in Table 7.

### 3.3. Exploratory Factor Analysis Findings

An exploratory factor analysis (EFA) was conducted to examine the construct validity of the TMJAQoL-TR scale. The suitability of the data for factor analysis was confirmed by a Kaiser–Meyer–Olkin (KMO) coefficient of 0.895, indicating excellent sampling adequacy. Bartlett’s test of sphericity was significant (χ^2^(66) = 624.63, *p* < 0.001), demonstrating that the correlations among items were sufficient for factor extraction.

Based on the Kaiser criterion (eigenvalue >1), a two-factor structure was obtained, accounting for 56.9% of the total variance. The first factor had an eigenvalue of 5.49 and explained 45.7% of the variance, while the second factor had an eigenvalue of 1.34 and explained 11.2% of the variance. After varimax rotation, items q6–q12 loaded strongly on Factor 1 (loading range: 0.60–0.85), and items q1–q5 loaded on Factor 2 (loading range: 0.53–0.70). Although item q5 demonstrated cross-loading, it was retained under Factor 2 due to its higher loading value (Table 8).

Overall, the analysis demonstrated that the TMJAQoL-TR scale operated on a two-dimensional structure consistent with clinical expectations.

### 3.4. Confirmatory Structural Modeling (AMOS)

**Figure 1:**
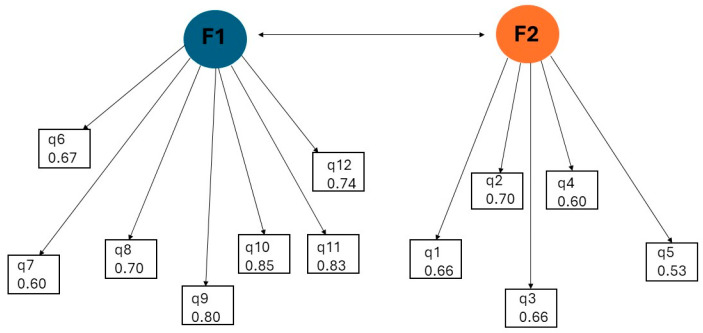
AMOS path diagram.

AMOS demonstrated a two-factor solution consistent with the theoretical structure of the TMJAQoL-TR (Figure 1), with acceptable standardized loadings (Table 9) and clinically interpretable factor covariance (0.48). Confirmatory factor analysis demonstrated an acceptable model fit for the two-factor structure of the TMJAQoL-TR. The analysis yielded the following fit indices: χ^2^(53) = 63.24, *p* = 0.158; χ^2^/df = 1.19; CFI = 0.958; TLI = 0.948; SRMR ≈ 0.155; RMSEA = 0.052. These values meet widely accepted psychometric criteria (CFI/TLI ≥0.90; RMSEA ≤0.06; χ^2^/df < 2), indicating that the proposed two-factor model demonstrates an adequate level of structural validity for this preliminary validation stage. The Standardized Root Mean Square Residual (SRMR) value above 0.10 indicates suboptimal global residual fit. Given the heterogeneous sample and limited ankylosis subgroup, this finding should be interpreted cautiously as a preliminary indication of model misfit, rather than definitive evidence against the underlying two-factor structure. Given the known sensitivity of SRMR to sample size variability and heterogeneous clinical composition, model evaluation emphasized incremental fit indices (CFI, TLI) and RMSEA, which are recommended as primary indicators in preliminary structural validation studies.

The figure below illustrates the two-factor structure of the TMJAQoL-TR, with latent variables (F1 and F2) loading on observed items and a covariance between factors.

Exploratory and confirmatory analyses supported a two-factor structure of the TMJAQoL-TR. Standardized factor loadings ranged from 0.53 to 0.85, exceeding the recommended minimum of 0.50 for all items (Table 10). The strongest loadings were observed for items q9–q11 (λ = 0.80–0.85), indicating a robust relationship with the functional limitation construct.

Communality (h^2^) values ranged from 0.28 to 0.72, demonstrating that the latent variables explained an acceptable proportion of item variance. No evidence of problematic cross-loading was identified, supporting factorial distinctiveness.

Construct reliability analysis demonstrated good internal consistency, with composite reliability values of 0.90 for Factor 1 and 0.77 for Factor 2. The average variance extracted was 0.56 for Factor 1 and 0.40 for Factor 2, indicating acceptable convergent validity, particularly considering the limited number of items in the second factor (Table 11).

These findings confirm that the TMJAQoL-TR exhibits a stable and interpretable latent structure in patients with severe temporomandibular disorders.

### 3.5. Ankylosis Subgroup Results

In the ankylosis subgroup, internal consistency of the TMJAQoL-TR remained acceptable, with a Cronbach’s alpha of 0.734. The mean age was 47.2 ± 13.4 years, and the median symptom duration was 54 months (range: 1–360). Maximal interincisal opening demonstrated a median of 33.5 mm (15–37). Lateral mandibular movements were restricted, with right deviation measuring 10 mm (5–20) and left deviation 8 mm (3–25).

Pain severity was moderate, with a mean VAS score of 4.6 ± 2.4. Quality of life outcomes indicated a clinically relevant impact, with a median TMJAQoL-TR score of 11.5 (7–25) and a median OHIP-14 score of 10 (1–35). SF-36 subscale values were as follows: physical function 62.9 ± 27.2, physical role 26.9 ± 39.3, pain 3.4 ± 1.1, general health 53.9 ± 9.1, vitality 55 ± 13.5, social function 51 ± 13.7, emotional role 38.2 ± 42.7, and mental health 59.3 ± 9.8. The median maximal interincisal opening was 33.5 mm (15–37), with no significant correlation with TMJAQoL-TR scores. The correlation between the TMJAQoL-TR questionnaire and the OHIP-14 was found to be high (*r* = 0.790; *p* < 0.01), while a moderate correlation was observed between the TMJAQoL-TR and the VAS pain scale (*r* = 0.449; *p* < 0.05). Additionally, a moderate and statistically significant correlation was found between the TMJAQoL-TR and the SF-36 general health subscale (*r* = 0.431; *p* < 0.05) (Table 12).

Due to the limited sample size in the ankylosis subgroup (n = 26), an independent exploratory factor analysis was not performed. Although the overall pattern of item clustering appeared conceptually compatible with the two-factor structure identified in the full sample, the subgroup size does not meet established methodological thresholds for factor extraction or stable eigenvalue estimation. Therefore, subgroup-level factor loadings are not reported to avoid overinterpretation. Instead, ankylosis results are presented descriptively (internal consistency, score distribution, item–scale correlations) to demonstrate preliminary structural coherence without claiming confirmatory evidence.

In summary, the factor structure was evaluated only in the total sample, and subgroup results are interpreted as supportive but non-conclusive due to sample size limitations.

Importantly, the ankylosis subgroup demonstrated measurement characteristics consistent with the overall sample, with internal consistency coefficients and score distributions showing no statistically relevant deviation from the broader cohort of patients with severe temporomandibular disorders (*p* > 0.05 across primary comparisons). These findings support the applicability of the TMJAQoL-TR in ankylosis while confirming that the subgroup does not behave psychometrically differently from the general sample at this exploratory stage.

## 4. Discussion

The results of this study demonstrate that the Turkish version of the TMJAQoL shows valid and reliable measurement properties in patients with TMJ ankylosis and in individuals with severe TMD-related functional limitations. To our knowledge, this is the first study to translate and culturally adapt the TMJAQoL into Turkish and to provide preliminary psychometric evidence in a sample consisting of both ankylosis patients and a clinically defined subgroup with comparable functional impairment. Furthermore, in line with the extended aim of the study, its validity and reliability were examined not only in patients with ankylosis but also in individuals with severe TMD-related functional limitations, who present comparable restriction in mouth opening, mastication, and daily activities. The statistical data obtained revealed that the scale exhibited high accuracy in terms of both internal consistency and test–retest reliability. Therefore, the present findings should be interpreted as preliminary and exploratory, rather than as a definitive condition-specific validation in ankylosis alone. Although 26 participants (21.7%) were diagnosed with TMJ ankylosis, the majority of the sample consisted of severe TMD cases without ankylosis. Therefore, potential bias in structural validity cannot be entirely excluded. The identified factor structure should be interpreted cautiously, particularly regarding its applicability to ankylosis-dominant populations.

The TMJAQoL-TR study was planned and implemented according to the required procedures. For translation and cultural adaptation, the method proposed by Beaton et al. [20] was used. The linguistic validity and cultural compatibility were found to be appropriate. The low floor and ceiling effect percentages support the scale’s ability to discriminate between varying levels of clinical severity, reducing the risk of score saturation and enhancing interpretability in both clinical follow-up and research applications.

The exploratory factor analysis demonstrated that the TMJAQoL-TR has a theoretically and clinically coherent two-factor structure, and this structure was subsequently supported by the AMOS structural model, which confirmed the distinction between functional impairment and symptom-related impact in individuals with temporomandibular disorders.

Confirmatory analysis provided preliminary support for the two-factor structure. Although the SRMR value exceeded conventional thresholds, incremental fit indices (CFI, TLI) and RMSEA indicated acceptable model fit. Considering the heterogeneous clinical composition of the sample and the relatively small ankylosis subgroup, model interpretation primarily relied on these more robust indices. Therefore, the structural findings should be regarded as preliminary and warrant confirmation in larger, diagnostically homogeneous samples.

The internal consistency reliability of the TMJAQoL-TR was strongly supported, with a Cronbach’s α coefficient exceeding 0.85; values above 0.80 are generally considered indicative of high reliability [33]. The ICC values for individual items were all above 0.90, demonstrating excellent agreement. Test–retest reliability analysis yielded ICC values ranging from 0.95 to 0.99, indicating a high level of temporal stability. Such high stability likely reflects the chronic and functionally stable nature of the condition rather than perfect measurement precision.

A high correlation (*r* = 0.772; *p* < 0.01) was found between the TMJAQoL-TR and the OHIP-14. The OHIP-14 is a widely used scale assessing the impact of oral health on overall quality of life [34]. This high correlation indicates that while the TMJAQoL-TR measures quality of life specifically in the context of TMJ ankylosis; it also produces results consistent with general oral-health-related quality of life measures. A moderate correlation (*r* = 0.312; *p* < 0.01) was observed between the TMJAQoL-TR and the VAS pain scale, which is a reliable tool for assessing patients’ subjective pain perception. This finding highlights the influence of TMJ ankylosis on patients’ perceived pain.

Correlation analyses with the SF-36 showed relationships between the TMJAQoL-TR and the subscales of physical function (*r* = −0.086), physical role (*r* = −0.212; *p* < 0.05), and emotional role (*r* = −0.084). The negative correlations, particularly with physical function, physical role, and emotional role, support the notion that TMJ ankylosis adversely affects individuals’ daily life activities.

In this study, the mean age of female patients was significantly higher than that of males (*p* = 0.004), suggesting that TMJ ankylosis may occur at later ages in women. Furthermore, the finding that 55% of the patients were housewives may indicate that the condition could limit participation in the workforce.

The mean maximal incisal opening was 43.7 ± 9.2 mm, with no significant correlation with TMJAQoL-TR scores. This finding suggests that a single linear measure of mouth opening may not fully capture patients’ perceived disability or psychosocial burden. However, right and left lateral deviation values (9.7 ± 3.9 mm and 9.6 ± 4.4 mm, respectively) showed moderate correlations with quality of life, supporting the notion that more complex functional disturbances in mandibular movements may be more closely aligned with patients’ subjective experiences. Among the most common problems faced by patients with TMJ ankylosis are chewing difficulties, speech impairments, and pain [10]. Our findings therefore indicate that the TMJAQoL-TR is effective in evaluating these aspects and accurately reflecting functional limitations relevant to daily life.

In the ankylosis subgroup, TMJAQoL-TR scores showed a clearer association with the SF-36 General Health subscale, whereas in the overall sample the strongest relationship was observed with the SF-36 Physical Role (role-physical) subscale. This difference may be related to the clinical profile of the groups: ankylosis often affects overall health perception due to long-term structural limitation, while severe TMD without ankylosis may interfere more with role-based physical activities. Despite this variation, other correlations—including age, symptom duration, pain scores, interincisal opening, and lateral mandibular movements—were similar between the subgroup and the total sample. These parallel patterns suggest that the TMJAQoL-TR performs consistently across both groups and reflects a shared functional impact rather than being dependent solely on diagnostic category. Nonetheless, subgroup differences should be interpreted cautiously and verified in future studies with larger ankylosis-specific samples.

The present findings support the structural validity of the TMJAQoL-TR, demonstrating a coherent two-factor model with satisfactory psychometric performance. All items showed adequate factor loadings and communalities, indicating meaningful representation of their respective constructs without redundancy. The absence of substantial cross-loadings suggests that the domains measure related but distinct aspects of TMJ-related quality of life.

Composite reliability values were within recommended ranges, confirming internal coherence of the constructs. Although the AVE for the second factor was slightly below the conventional 0.50 criterion, this is acceptable for multidimensional clinical scales with relatively few items and heterogeneous symptom profiles. Similar findings have been reported in cross-cultural adaptations of condition-specific quality of life instruments.

Importantly, the factor structure reflects not only ankylosis-related disability but also the broader clinical spectrum of severe TMD, which may explain minor deviations from the original scale configuration. Therefore, the TMJAQoL-TR should be interpreted as a valid instrument for assessing TMJ-related quality of life across severe TMJ pathologies rather than ankylosis alone.

COSMIN evaluation demonstrated sufficient evidence for content validity, internal consistency, reliability, and construct validity of the TMJAQoL-TR, supporting its use as a culturally adapted patient-reported outcome measure in individuals with severe temporomandibular disorders. From a clinical perspective, the TMJAQoL-TR can be used as a complementary tool for treatment planning and outcome monitoring. Repeated administrations over time can be used to document changes following surgical correction or conservative therapy and to facilitate shared decision-making with patients.

### Limitations

The primary limitation of this study is the heterogeneous clinical composition of the sample, including a relatively small number of patients with confirmed temporomandibular joint ankylosis. This may limit the generalizability of findings to ankylosis-specific populations. Additionally, diagnostic procedures were not fully standardized across all participants, and not all patients underwent advanced imaging, which may have introduced potential classification bias.

The total sample size, while adequate for preliminary validation, may be considered borderline for confirmatory factor analysis. Therefore, the structural findings should be interpreted as preliminary and require replication in larger, diagnostically homogeneous cohorts.

Attrition bias cannot be fully excluded, as baseline characteristics of participants who completed the retest were not formally compared with those who did not. Furthermore, responsiveness was not evaluated, and longitudinal studies are needed to assess sensitivity to clinical change.

## 5. Conclusions

The TMJ Ankylosis Quality of Life Questionnaire has been translated into Turkish and its preliminary psychometric evaluation has been carried out in this study. The TMJAQoL-TR has proven to be valid and reliable in a group of patients suffering from extreme functional impairment due to temporomandibular joint-related issues.

The main limitations of this study were the heterogeneous nature of the study population and the relatively small number of patients with confirmed TMJ ankylosis. However, subgroup analyses still alluded to comparable psychometric properties in ankylosis patients and in the overall population. Thus, it can be concluded that TMJAQoL-TR may serve as a patient-reported outcome measure for quality of life assessment in severe TMD, including ankylosis.

To validate these results and to further solidify the clinical applicability of the scale more extensive, structured studies will be needed that consist of large, homogeneous ankylosis-specific populations and multicenter designs.

## Figures and Tables

**Table 1:** Demographic variables (n = 120).

Variable	n (%)
Gender	
Female	88 (73.3%)
Male	32 (26.7%)
Education	
Primary school	68 (56.7%)
High school	23 (19.2%)
University	29 (24.2%)
Occupation	
Housewife	66 (55.0%)
White collar	23 (19.2%)
Blue collar	15 (12.5%)
Student	4 (3.3%)
Retired	12 (10.0%)

**Table 2:** General medical conditions of the participants (n = 120).

Comorbidity	
None	84 (70.0%)
Present	36 (30.0%)
– Hypertension	15 (41.7%)
– Fibromyalgia	6 (16.7%)
– Diabetes mellitus	5 (13.9%)
– Ankylosing spondylitis	4 (11.1%)
– Rheumatoid arthritis	2 (5.6%)
– Psoriatic arthritis	1 (2.8%)
– Hypothyroidism	1 (2.8%)
– Sarcoidosis	1 (2.8%)
– Peptic ulcer	1 (2.8%)

**Table 3:** Types of temporomandibular joint ankylosis according to Sawhney’s classification (n = 26).

TMJ Ankylosis Type	Movement Limitation
Type 1	12
Type 2	9
Type 3	3
Type 4	2

**Table 4:** Scores of the evaluation scales applied to patients.

Parameter	Mean ± SD	Median	Min–Max
TMJAQoL-TR	9.4 ± 7.7	8	0–45
OHIP-14	11.2 ± 8.1	10	0–36
Maximal incisal opening (mm)	43.7 ± 9.2	43.5	15–65
Right deviation (mm)	9.7 ± 3.9	10	2–20
Left deviation (mm)	9.6 ± 4.4	10	0–25
VAS pain score	4.7 ± 2.9	5	0–10
SF-36—Physical function	61.4 ± 25.3	60	0–100
SF-36—Physical role	39.5 ± 41.9	25	0–100
SF-36—Pain	3.3 ± 1.1	3.5	1–6
SF-36—General health	54.2 ± 9.8	55	30–85
SF-36—Vitality	50 ± 14.2	50	5–80
SF-36—Social function	49.8 ± 13.1	50	13–88
SF-36—Emotional role	44.6 ± 40.6	33.3	0–100
SF-36—Mental health	58.6 ± 12.7	60	0–100

TMJAQoL-TR: Turkish version of Temporomandibular Joint Ankylosis Quality of Life, OHIP-14: Oral Health Impact Profile Short Form-14, mm: millimeter, VAS: Visual Analog Scale, SF-36: Short Form-36, SD: Standard Deviation, Min: Minimum, Max: Maximum.

**Table 5:** Item-based reliability profile including internal consistency, test–retest ICC, and Weighted Cohen’s Kappa Agreement.

	Cronbach’s Alpha If Item Deleted	Retest ICC (95% CI)	Weighted Cohen’s Kappa
item 1	0.887	0.984 (0.974–0.990)	0.941
item 2	0.878	0.992 (0.987–0.995)	0.976
item 3	0.871	0.993 (0.989–0.996)	0.979
item 4	0.866	0.964 (0.942–0.977)	0.896
item 5	0.871	0.971 (0.954–0.982)	0.917
item 6	0.867	0.987 (0.979–0.992)	0.956
item 7	0.866	0.982 (0.971–0.989)	0.940
item 8	0.861	0.972 (0.956–0.983)	0.883
item 9	0.861	0.954 (0.927–0.971)	0.835
item 10	0.865	0.991 (0.986–0.995)	0.976
item 11	0.870	0.991 (0.986–0.994)	0.971
item 12	0.865	0.993 (0.989–0.996)	0.980

**Table 6:** Measurement error indices for TMJAQoL-TR and factors.

Total/Factor	SD	ICC	SEM	MDC_95_
Total	7.677	0.995	0.54	1.5
Factor 1—Function and Pain	4.853	0.995	0.34	0.94
Factor 2—Symptoms and Daily Life	3.713	0.988	0.41	1.14

TMJAQoL-TR: Turkish version of Temporomandibular Joint Ankylosis Quality of Life, SD: Standard Deviation, ICC: Intraclass Correlation Coefficient, SEM: Standard Error of Measurement, MDC_95_: Minimal Detectable Change at the 95% confidence level.

**Table 7:** Correlations between assessment parameters.

Variables	TMJAQoL-TR	OHIP-14	VAS Pain	Physical Function	Physical Role	Pain	General Health	Vitality	Social Function	Emotional Role	Mental Health
TMJAQoL-TR	1.000	0.772 **	0.312 **	−0.086	−0.212 *	0.096	0.029	0.001	0.102	−0.084	0.002
OHIP-14	0.772 **	1.000	0.370 **	−0.031	−0.222 *	0.152	0.043	0.045	0.121	−0.111	−0.030
VAS Pain	0.312 **	0.370 **	1.000	−0.239 **	−0.324 **	–	0.040	−0.067	−0.030	−0.141	−0.131
Physical Function	−0.086	−0.031	−0.239 **	1.000	0.619 **	−0.584 **	−0.194 *	−0.095	−0.074	0.372 **	0.077
Physical Role	−0.212 *	−0.222 *	−0.324 **	0.619 **	1.000	−0.635 **	−0.282 **	−0.077	−0.202 *	0.561 **	0.012
Pain	0.096	0.152	0.333 **	−0.584 **	−0.635 **	1.000	0.293 **	0.015	0.056	−0.361 **	−0.088
Emotional Role	−0.084	−0.111	−0.141	0.372 **	0.561 **	−0.361 **	−0.036	−0.060	−0.173	1.000	−0.036

* Correlation is significant at the 0.05 level (2-tailed), ** Correlation is significant at the 0.01 level (2-tailed). TMJAQoL-TR: Turkish version of Temporomandibular Joint Ankylosis Quality of Life, OHIP-14: Oral Health Impact Profile Short Form-14, VAS: Visual Analog Scale.

**Table 8:** Factor loadings after varimax rotation.

Item	Factor 1	Factor 2
q1	—	0.66
q2	—	0.70
q3	—	0.66
q4	—	0.60
q5	0.40	0.53
q6	0.67	—
q7	0.60	—
q8	0.70	—
q9	0.80	—
q10	0.85	—
q11	0.83	—
q12	0.74	—

Conceptually, the two obtained factors were interpreted as: Factor 1: Temporomandibular Function and Pain, Factor 2: Symptoms and Daily Life Impact.

**Table 9:** Standardized regression weights.

Path	Std. Estimate
F1 → q6	0.67
F1 → q7	0.60
F1 → q8	0.70
F1 → q9	0.80
F1 → q10	0.85
F1 → q11	0.83
F1 → q12	0.74
F2 → q1	0.66
F2 → q2	0.70
F2 → q3	0.66
F2 → q4	0.60
F2 → q5	0.53

**Table 10:** Factor loadings, communalities, and construct reliability of the TMJAQoL-TR.

Item	Factor	Standardized Loading (λ)	Communality (h^2^)
q1	F2	0.66	0.44
q2	F2	0.70	0.49
q3	F2	0.66	0.44
q4	F2	0.60	0.36
q5	F2	0.53	0.28
q6	F1	0.67	0.45
q7	F1	0.60	0.36
q8	F1	0.70	0.49
q9	F1	0.80	0.64
q10	F1	0.85	0.72
q11	F1	0.83	0.69
q12	F1	0.74	0.55

TMJAQoL-TR: Turkish version of Temporomandibular Joint Ankylosis Quality of Life Questionnaire, λ: standardized factor loading, h^2^: communality.

**Table 11:** Construct-level metrics.

Factor	No. Items	Composite Reliability (CR)	AVE
F1	7	0.90	0.56
F2	5	0.77	0.40

CR: composite reliability, AVE: average variance extracted.

**Table 12:** Correlations between assessment parameters (Ankylosis Subgroup Results: n = 26).

Variables	OHIP-14	VAS Pain	Physical Function	Physical Role	Pain	General Health	Vitality	Social Function	Emotional Role	Mental Health
TMJAQoL-TR	0.790 **	0.449 *	0.089	0.057	0.116	−0.431 *	−0.015	−0.001	0.162	−0.035

* Correlation is significant at the 0.05 level (2-tailed), ** Correlation is significant at the 0.01 level (2-tailed). TMJAQoL-TR: Turkish version of Temporomandibular Joint Ankylosis Quality of Life, OHIP-14: Oral Health Impact Profile Short Form-14, VAS: Visual Analogue Scale.

## Data Availability

The raw data supporting the conclusions of this article will be made available by the authors on request.

## Supplementary Materials

The following supporting information can be downloaded at: https://www.mdpi.com/article/10.3390/healthcare14050644/s1, File S1: Expanded COSMIN Risk of Bias Assessment for the TMJAQoL-TR Validation Study; Figure S1: Study Flow Diagram.

## Abbreviations

CBCT	Cone Beam Computed Tomography
DC/TMD	Diagnostic Criteria for Temporomandibular Disorders
EFA	Exploratory factor analysis
ICC	The Intraclass Correlation Coefficient
KMO	Kaiser–Meyer–Olkin
Max	Maximum
Min	Minimum
Mm	Millimeter
MRI	Magnetic Resonance Imaging
OBC-21	Oral Behaviors Checklist-21
OHIP-14	Oral Health Impact Profile Short Form-14
OMUKAEK	Ondokuz Mayıs University Clinical Research Ethics Committee
OQoLQ	Orthognathic Quality of Life Questionnaire
SD	Standard Deviation
SF-36	Short Form-36
TMD	Temporomandibular Disorders
TMJ	Temporomandibular Joint
TMJAQoL	Temporomandibular Joint Ankylosis Quality of Life
TMJAQoL-TR	Turkish version of Temporomandibular Joint Ankylosis Quality of Life
VAS	Visual Analog Scale
WHO	World Health Organization
