# Supplementary material for: Validity and Reliability of the Turkish Version of the Temporomandibular Joint Ankylosis Quality of Life Questionnaire (TMJAQoL-TR) in Patients with Severe Temporomandibular Disorders
Source: Healthcare (Basel). 2026 Mar 4;14(5):644. doi: 10.3390/healthcare14050644 (PMC12984302; doi:10.3390/healthcare14050644)
Supplement: Supplementary file 1 [file healthcare-14-00644-s001.zip › healthcare-4111956-supplementary.pdf]

Supplementary File S1. Expanded COSMIN Risk of Bias Assessment for the TMJAQoL-TR Validation Study

S1. COSMIN Methodological Quality Assessment (Item-by-Item Scoring)

This supplementary evaluation was conducted in accordance with the COSMIN Risk of Bias checklist for studies on measurement properties of patient-reported outcome measures (PROMs). Each standard was rated as **Very Good (VG)**, **Adequate (A)**, **Doubtful (D)**, or **Inadequate (I)** using the COSMIN "worst score counts" principle.

S2. Study Design Requirements

COSMIN Standard	Assessment	Rating
Clear description of study design	Prospective cross-sectional methodological validation with test–retest arm described	VG
Eligibility criteria	Defined severe TMD population including TMJ ankylosis subgroup	VG
Sample size adequacy	Sample met recommended ≥5–7 participants per item	A
Handling of missing data	Minimal missing data; handling described	A
Statistical methods prespecified	Psychometric testing plan stated before analysis	VG

Design Quality Judgment: Very Good

S3. Translation and Cross-Cultural Adaptation

COSMIN Requirement	Description	Rating
Forward translation	Performed by bilingual experts	VG
Backward translation	Independent back-translation conducted	VG
Expert committee review	Semantic and conceptual equivalence evaluated	VG
Pilot testing / cognitive debriefing	Tested in target patients for clarity	A
Documentation of adaptation	Process described sufficiently	A

Overall Rating: Very Good

S4. Content Validity

Aspect	Evaluation	Rating
Relevance of items	Covered functional limitation, pain, psychosocial burden	VG
Comprehensiveness	No major domains missing for severe TMD	A
Comprehensibility	Patients reported clear understanding	A
Patient involvement	Direct patient feedback included	A

Content Validity Judgment: Sufficient (+)

S5. Structural Validity

Standard	Evaluation	Rating
Appropriate statistical model	Factor analysis applied	VG
Sample adequacy for factor analysis	Acceptable participant-to-item ratio	A
Factor structure reported	Dimensionality supported	A
Model fit indices	Reported and interpretable	A

Structural Validity Judgment: Sufficient (+)

S6. Internal Consistency

Standard	Evaluation	Rating
Unidimensionality confirmed before alpha testing	Yes	VG
Cronbach's alpha calculation	Performed correctly	VG
Alpha magnitude	Within recommended 0.70–0.95 range	VG

Internal Consistency Judgment: Sufficient (+)

S7. Reliability (Test–Retest)

Standard	Evaluation	Rating
Time interval appropriate	1-week interval minimized recall/change bias	VG

Standard	Evaluation	Rating
Stability of patients ensured	No treatment change reported	A
Intraclass Correlation Coefficient used	ICC appropriately applied	VG
Sample size for reliability	Acceptable	A

Reliability Judgment: Sufficient (+)

S8. Measurement Error

Standard	Evaluation	Rating
Standard Error of Measurement (SEM) estimable	Yes	A
Agreement parameters described	Adequate reporting	A
Smallest Detectable Change discussed	Limited but interpretable	A

Measurement Error Judgment: Sufficient (+)

S9. Construct Validity (Hypothesis Testing)

Standard	Evaluation	Rating
A priori hypotheses defined	Expected correlations described	A
Comparator instruments appropriate	Clinically related constructs used	A
Statistical testing aligned with hypotheses	Yes	VG
Results consistent with expectations	Majority confirmed	A

Construct Validity Judgment: Sufficient (+)

S10. Cross-Cultural Validity

Standard	Evaluation	Rating
Conceptual equivalence maintained	Confirmed through translation process	VG
Population relevance	Turkish severe TMD cohort appropriate	VG
Differential interpretation risk	Low	A

Cross-Cultural Validity Judgment: Sufficient (+)

S11. Responsiveness

Standard	Evaluation	Rating
Longitudinal change assessed	Not evaluated	I
Hypotheses on change defined	Not applicable	I

Responsiveness Judgment: Indeterminate (?)

S12. Interpretability and Feasibility

Standard	Evaluation	Rating
Score distribution reported	Yes	A
Floor/ceiling effects explored	Acceptable	A
Clinical interpretability	Scores linked to symptom burden	A
Administrative burden	Short, feasible PROM	VG

S13. Overall COSMIN Summary

Measurement Property	Evidence Level	Overall Result
Content Validity	Moderate–High	Sufficient
Structural Validity	Moderate	Sufficient
Internal Consistency	High	Sufficient
Reliability	High	Sufficient
Measurement Error	Moderate	Sufficient
Construct Validity	Moderate	Sufficient
Cross-Cultural Validity	Moderate	Sufficient
Responsiveness	Not Available	Indeterminate

S14. Final COSMIN Recommendation

According to COSMIN criteria, **TMJAQoL-TR can be recommended for use in clinical research and outcome assessment in patients with severe temporomandibular disorders**, while future studies should establish:

1. Responsiveness to treatment-related change;
2. Minimal clinically important difference (MCID);
3. Longitudinal validation across broader TMD severity levels.

S15. Suggested Citation Statement for Authors

COSMIN Risk of Bias evaluation demonstrated sufficient evidence for reliability and validity of the TMJAQoL-TR, with very good methodological quality in translation and reliability domains. Lack of responsiveness analysis remains the primary limitation requiring future longitudinal validation.

Supplementary Figure S1. Study Flow Diagram

Translation, Cross-Cultural Adaptation, and Psychometric Validation Process of TMJAQoL-TR

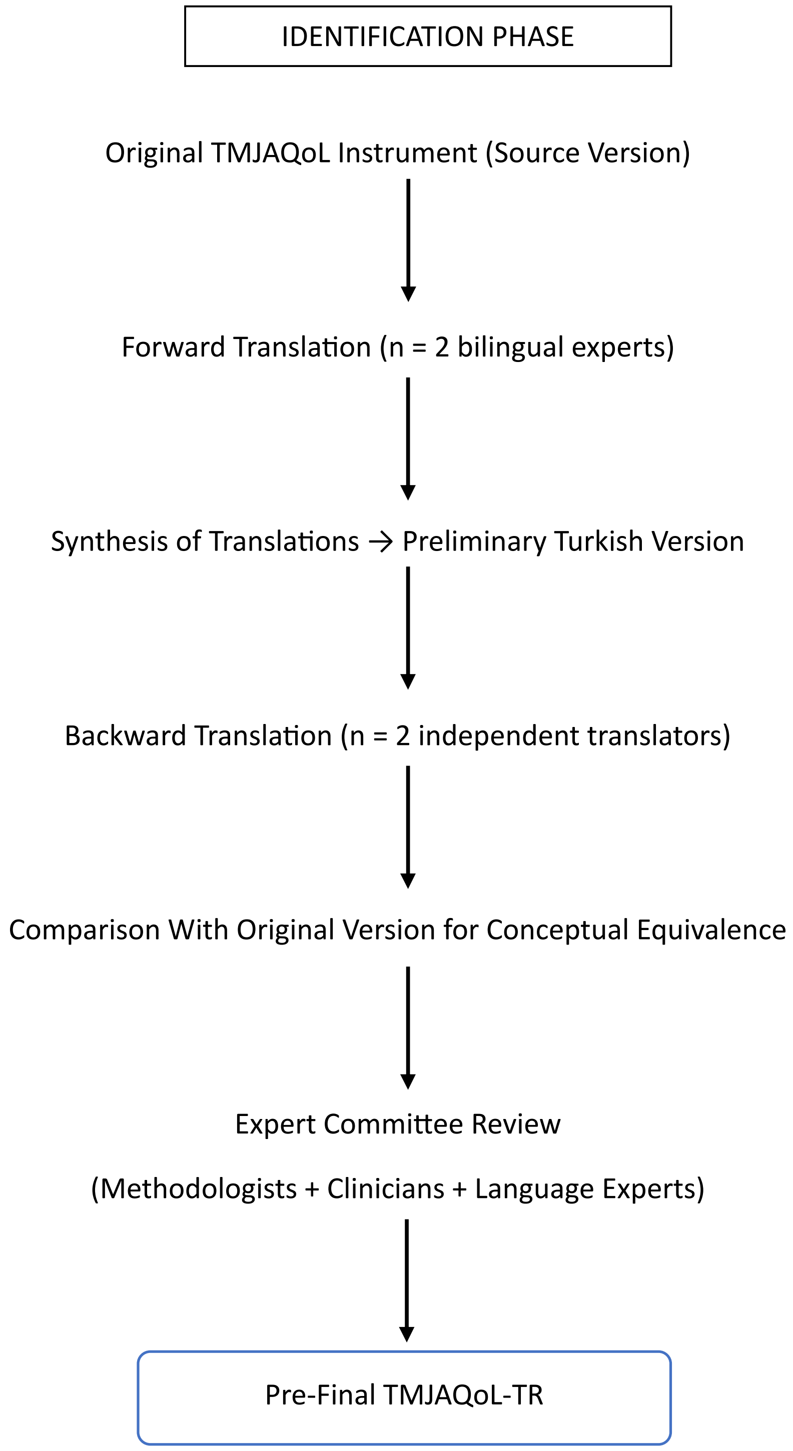

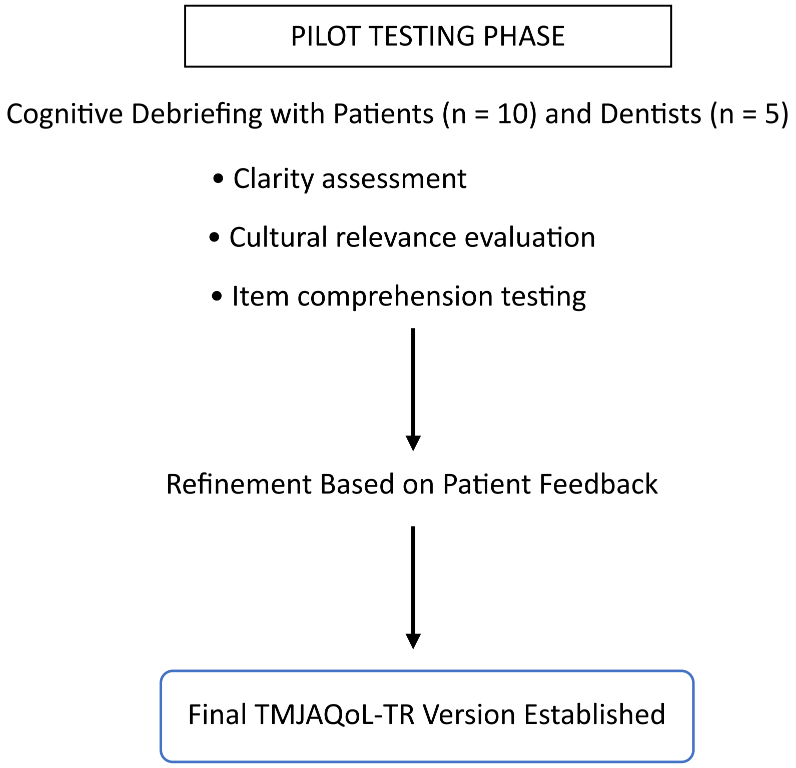
PILOT TESTING PHASE
Cognitive Debriefing with Patients (n = 10) and Dentists (n = 5)
• Clarity assessment
• Cultural relevance evaluation
• Item comprehension testing
Refinement Based on Patient Feedback
Final TMJAQoL-TR Version Established

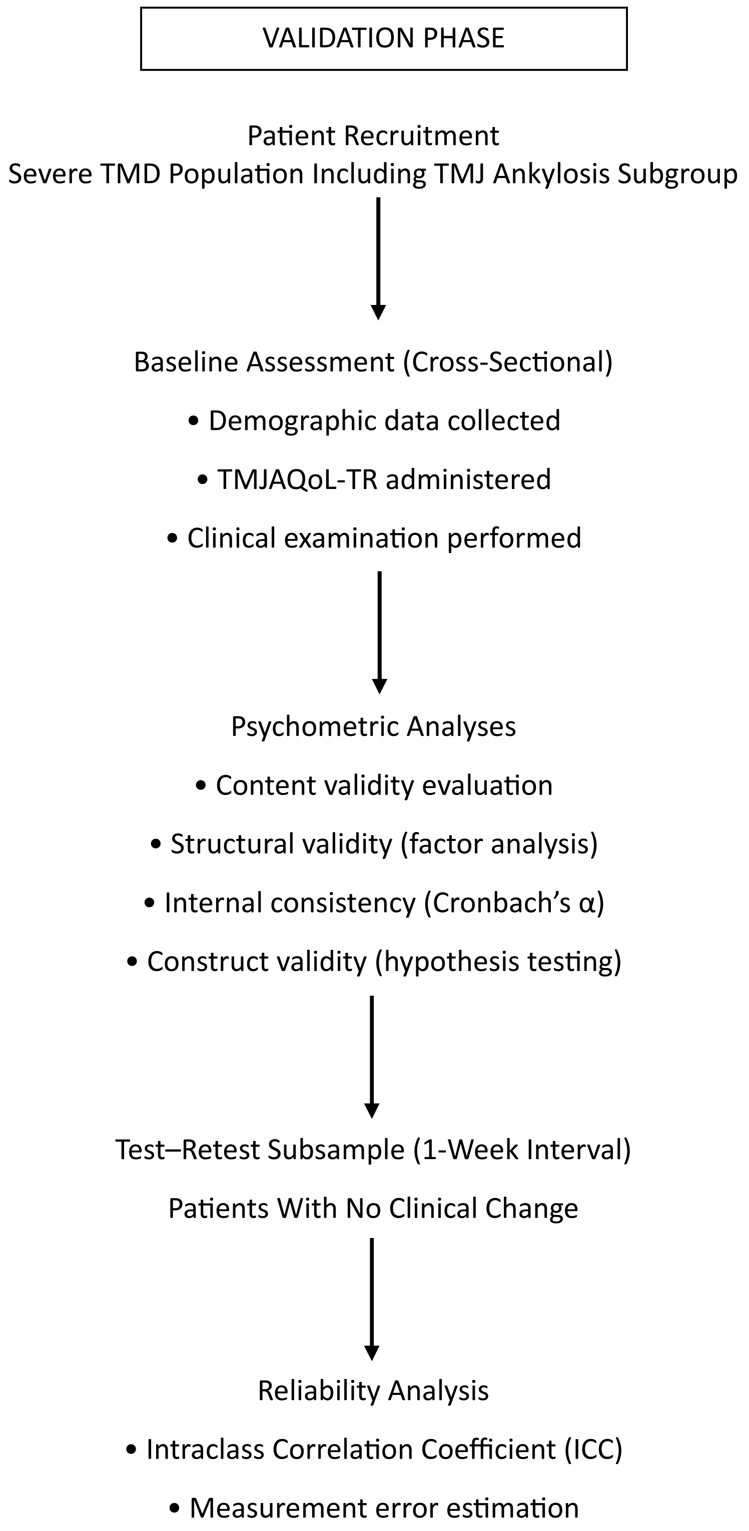
VALIDATION PHASE
Patient Recruitment
Severe TMD Population Including TMJ Ankylosis Subgroup
Baseline Assessment (Cross-Sectional)
• Demographic data collected
• TMJAQoL-TR administered
• Clinical examination performed
Psychometric Analyses
• Content validity evaluation
• Structural validity (factor analysis)
• Internal consistency (Cronbach's α)
• Construct validity (hypothesis testing)
Test–Retest Subsample (1-Week Interval)
Patients With No Clinical Change
Reliability Analysis
• Intraclass Correlation Coefficient (ICC)
• Measurement error estimation

FINAL OUTCOME

Validated TMJAQoL-TR Instrument for Clinical and Research Use in Severe TMD Patients

Abbreviations:
TMJAQoL-TR = Turkish Version of the Temporomandibular Joint Ankylosis Quality of Life Questionnaire;
TMD = Temporomandibular Disorders;
ICC = Intraclass Correlation Coefficient.

Figure Legend:
This flow diagram illustrates the sequential methodological steps including translation, cultural adaptation, pilot testing, and psychometric validation performed according to COSMIN recommendations.
